# Arthroscopic Centralization of the Medial Meniscus Reduces Load on a Posterior Root Repair Under Dynamic Varus Loading: A Biomechanical Investigation

**DOI:** 10.1177/03635465241274791

**Published:** 2024-09-15

**Authors:** Adrian Deichsel, Christian Peez, Michael J. Raschke, R. Geoff Richards, Boyko Gueorguiev, Ivan Zderic, Elmar Herbst, Christoph Kittl

**Affiliations:** † Department of Trauma, Hand and Reconstructive Surgery, University Hospital Münster, Münster, Germany; ‡ AO Research Institute Davos, Davos, Switzerland; Investigation performed at Department of Trauma, Hand and Reconstructive Surgery, University Hospital Münster, Münster, Germany

**Keywords:** medial meniscus, meniscotibial ligament, extrusion, centralization, alignment

## Abstract

**Background:**

In addition to the integrity of the meniscal hoop function, both the anterior and posterior meniscus roots as well as the meniscotibial and meniscofemoral ligaments are crucial in restraining meniscal extrusion. However, the interaction and load sharing between the roots and these peripheral attachments (PAs) are not known.

**Purposes:**

To investigate the influence of an insufficiency of the PAs on the forces acting on a posterior medial meniscus root repair (PMMRR) in both neutral and varus alignment and to explore whether meniscal centralization reduces these forces.

**Study Design:**

Controlled laboratory study.

**Methods:**

In 8 fresh-frozen human cadaveric knees, an arthroscopic transosseous root repair (step 1) was performed after sectioning the posterior root of the medial meniscus. The pull-out suture was connected to a load cell to allow measurement of the forces acting on the root repair. A medial closing-wedge distal femoral osteotomy was performed to change the mechanical axis from neutral to 5° of varus alignment. The meniscus was completely released from its PAs (step 2), followed by transosseous arthroscopic centralization (step 3). Each step was tested in both neutral and varus alignment. The specimens were subjected to nondestructive dynamic varus loading under axial compression of 300 N in 0°, 15°, 30°, 45°, and 60° flexion. The changes in force acting on the PMMRR were statistically analyzed using a mixed linear model.

**Results:**

Axial loading in neutral alignment led to an increase of the force of root repair of 3.1 ± 3.1 N (in 0° flexion) to 6.3 ± 4.4 N (in 60° flexion). In varus alignment, forces increased significantly from 30° (3.5 N; 95% CI, 1.1-5.8 N; *P* = .01) to 60° (7.1 N; 95% CI, 2.7-11.5 N; *P* = .007) flexion, in comparison with neutral alignment. Cutting of the PAs in neutral alignment led to a significant increase of root repair forces in all flexion angles, from 7.0 N (95% CI, 1.0-13.0 N; *P* = .02) to 9.1 N (95% CI, 4.1-14.1 N; *P* = .003), in comparison with the intact state. Varus alignment significantly increased the forces in the cut states from 4.8 N (95% CI, 1.0-8.5 N; *P* = .02) to 11.1 N (95% CI, 4.2-18.0 N; *P* = .006) from 30° to 60° flexion, in comparison with the neutral alignment. Arthroscopic centralization led to restoration of the native forces in both neutral and varus alignment, with no significant differences between the centralized and intact states.

**Conclusion:**

An insufficiency of the PAs of the medial meniscus, as well as varus alignment, led to increased forces acting on a PMMRR. These forces were reduced via an arthroscopic meniscal centralization.

**Clinical Relevance:**

Performing arthroscopic meniscal centralization concomitantly with PMMRR may reduce failure of the repair by reducing the load of the root.

Tears of the posterior medial meniscus root (PMMR) are frequent and are known to increase medial compartment pressure and predispose to development of osteoarthritis.^1,30,36,41,43,44^ A PMMR repair (PMMRR) effectively restores cartilage biomechanics but is related to high failure rates, with numerous reasons for them discussed in the literature, most notably leg axis malalignment.^8,20,60^

Meniscal extrusion, defined as a meniscus being pressed out of its compartment, is a phenomenon associated with failure of a meniscal repair and a progression of osteoarthritic changes of the knee.^5,8,19^ Besides root tears, other types of meniscal tears that cause meniscal hoop dysfunction or meniscal resection could lead to extrusion.^18,45^ In addition to the roots, the medial meniscus is suspended by the peripheral attachments (PAs), composed of the circumferential meniscotibial ligaments (MTLs; also known as coronary ligaments) and meniscocapsular attachments (MCAs).^52,54,56^ Recently, the PAs were discussed as possible contributors to meniscal stability. Insufficiency of the PAs was shown to lead to extrusion of the meniscus, independent of the root integrity, in both clinical and biomechanical studies.^11,28,46^ To retract an extruded meniscus back into the tibiofemoral compartment, various techniques of meniscal centralization have been established,^9,25,26^ resulting in improved clinical and radiographic outcomes.^24,29^ As the meniscus roots and PAs interact to prevent meniscal extrusion, these surgical procedures simulating the function of the MTLs may reduce the forces acting on a root repair and thus potentially decrease the risk of clinical failure.

Therefore, the aims of the present study were to investigate the influence of an insufficiency of the PA on the forces acting on a PMMRR in both neutral and varus alignment and to explore whether arthroscopic meniscal centralization may decrease these forces. It was hypothesized that, whereas an insufficiency of the PA increases the forces acting on a PMMRR, these forces would be decreased after meniscal centralization.

## Methods

### Specimens and Preparation

Eight unpaired fresh-frozen human cadaveric knee specimens (age 73.0 ± 9.5 years (mean value ± standard deviation); 8 male; 5 right, 3 left), with no evidence of osteoarthritis, meniscal defects, other known injury of the knee, or previous knee surgery, were obtained from an international tissue bank (Science Care). During their lifetime, the donors bequeathed their corpse for use in medical science. Written consent was obtained from all donors such that no local or national ethics approval was required. After testing, each knee was dissected to check for ligamentous or meniscal injuries not specified in the testing protocol.

Specimens were thawed for 24 hours at room temperature before preparation. The femur and tibia were cut 250 mm above and below the joint line and embedded in aluminum tubes using polymethylmethacrylate (Suter Kunststoffe AG) such that the tibia and femur were centralized with the longitudinal tube axis aligned through the center of the intercondylar eminence and femoral notch.^3,57^ The skin and subcutaneous tissue were resected, leaving fascia and muscles intact. The fibula was then cut 100 mm distal to the proximal tibiofibular joint and transfixed with a 3.5-mm cortical screw to the tibia.^48^ Specimens were wrapped in tissue paper soaked with normal saline (0.9% NaCl) to prevent drying.

### Biomechanical Testing

**Figure 1.**
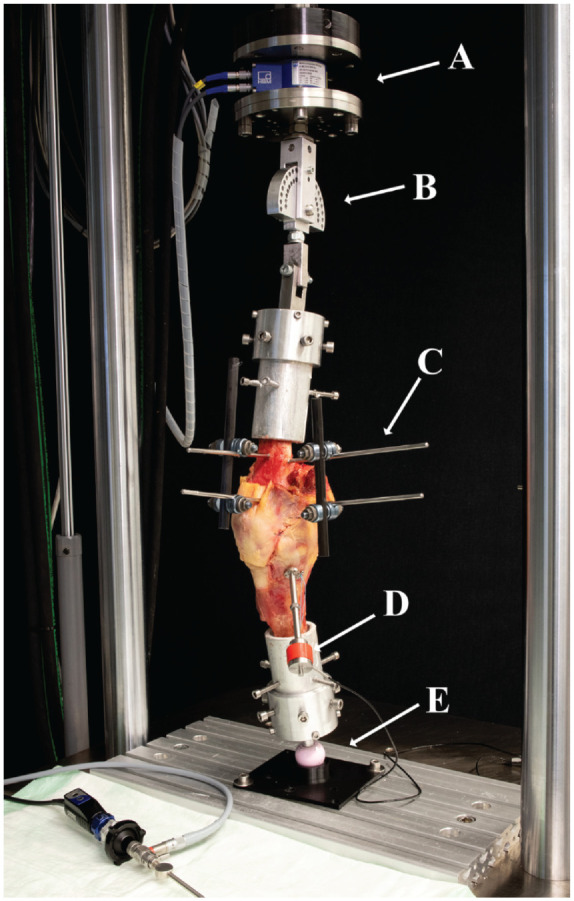
Test setup with a specimen mounted for biomechanical testing. (A) Load cell of the servohydraulic testing machine. (B) Rotating joint allowing flexion/extension and varus/valgus. (C) External fixator for fixation of medial closing-wedge osteotomy. (D) Tensioning device with load cell. (E) Ball-and-socket joint allowing flexion/extension, varus/valgus, and rotation.

Biomechanical testing was performed on a servohydraulic material testing machine (Bionix 858.20; MTS Systems Corp) equipped with a 5-kN load cell (HUPPERT 6; HUPPERT GmbH). The testing machine was coupled with a custom-made rig (Figure 1). The femoral end of the specimen was attached to the traverse of the testing machine in a fashion allowing for a fixed flexion angle of the knee joint and free varus/valgus angulation. The tibial end of the specimen allowed for free flexion/extension, rotation, and angulation, using a ball and socket joint. Under axial compression, the test rig allowed dynamic varus, which was defined as a laterally directed shift of the knee under axial compression. For every test sequence, the knee was loaded with an axial force from 0 to 300 N in 0°, 15°, 30°, 45°, and 60° flexion (1 loading cycle per flexion angle).

### Measurement of Meniscus Root Forces

**Figure 2.**
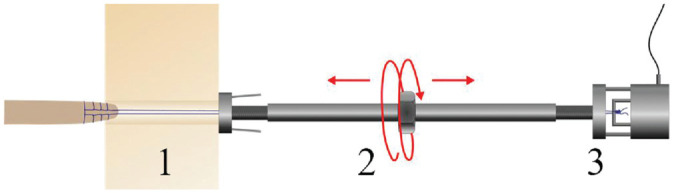
Custom-made tensioning and force measurement device. The suture (1) of the posterior medial meniscus root repair is shuttled through the device and fixed to a load cell (3). Tensioning of the suture is achieved by turning the center nut (2), which telescopes the threads out of the device.

After detaching the posterior root of the medial meniscus from its tibial insertion site, a transosseous repair was performed as previously described.^17^ The root was sutured using a suture passer (FIRSTPASS MINI; Smith & Nephew), with a high-strength polyethylene suture (No. 2 FiberWire; Arthrex) in a single-loop technique.^16^ A 2.0-mm K-wire was placed in the center of the anatomic posterior medial root tibial insertion site using a tibial aiming guide (Karl Storz), which was then overdrilled using a 4.5-mm cannulated drill bit up to the subchondral bone to avoid damage to the articular cartilage. The transosseous pull-out suture was shuttled through the bone tunnel and into a previously described custom-made tensioning device (Figure 2).^47^ Measurement of the forces acting on the suture was realized by fixing the suture to a load cell connected to the tensioning device (Y1 in-line threaded force transducer; Flintec GmbH), which was connected over an amplifier (DAD141.1; Flintec GmbH) to a data acquisition device (USB 6343; National Instruments), which was connected to a laptop running a custom-made LabVIEW (LabVIEW 2020; National Instruments) script. The load cell was calibrated by incrementally applying tensile loads from 0 to 200 N while recording the voltage output, to create a calibration curve. Tensioning of the suture was achieved through turning the center nut, which telescoped the threads out of the device. Pilot testing demonstrated tensile forces of 20 N acting on the transtibial pull-out root repair when the sutures were tied over a cortical button, being comparable to the shear forces acting on the PMMR during bipedal stance.^12,21,53,59^ Therefore, the preload of the root repair was set to 20 N before each dynamic loading cycle of the specimen, as specified above. The change of force acting on the transosseous root repair under dynamic loading was recorded for each load cycle.

### Varisation Distal Femoral Osteotomy

**Figure 3.**
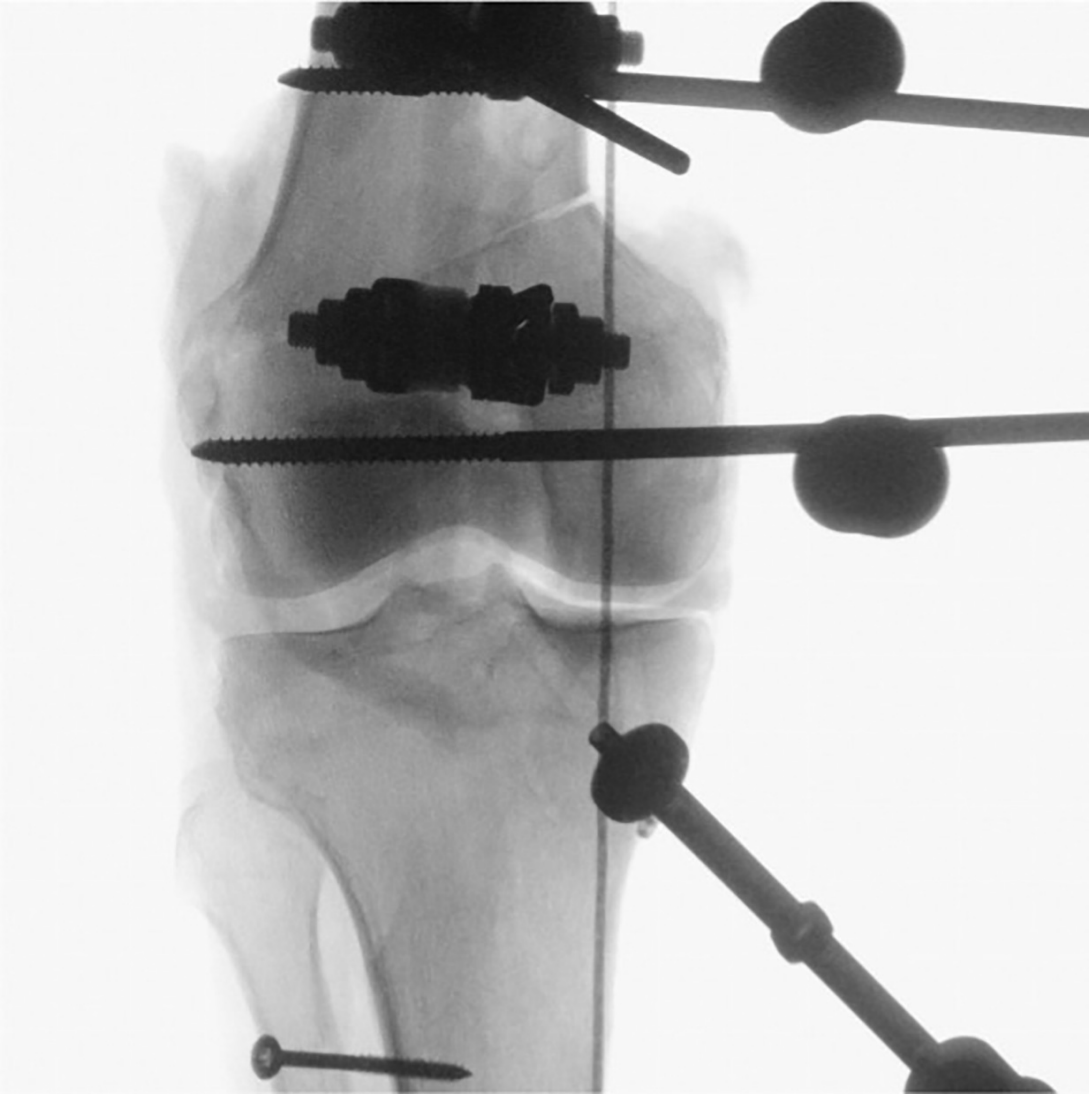
Anterior-posterior radiograph after medial closing-wedge distal femoral osteotomy to create varus alignment of the knee. The correct varus alignment was defined by the cable crossing the medial tibial plateau at 25% of the total tibial plateau width, corresponding to 5° of mechanical axis varus.

As previously described, a medial closing-wedge distal femoral osteotomy was performed using the technique described by Wylie and Mak^58^ to gradually adjust the alignment of each specimen and thus to evaluate the influence of varus alignment on the PMMRR forces.^47^ Under radiographic control, the osteotomy plane was marked by drilling 2 parallel 2.4-mm K-wires through the distal femoral metaphysis starting 10 mm proximal to the medial epicondyle. The tips of the K-wires were placed at the upper border of the lateral femoral epicondyle marking the safe zone of the lateral hinge.^23^ Two further 2.4-mm K-wires were placed 10 mm proximally, to mark the wedge to be taken out, to create varisation of the axis. Then, the osteotomy was performed using an oscillating saw, and the bone wedge was extracted, taking care to maintain an intact lateral hinge of 10 mm in width. The osteotomy was performed in the supracondylar metaphysis of the distal femur to avoid changes in the length and thus in the tension of the surrounding ligamentous structures. Under radiographic control, two 4.5-mm Steinmann pins were drilled into the medial distal femur 15 mm proximal and distal to the osteotomy plane in the mediolateral direction. The osteotomy was stabilized using an external fixator, mounted onto the Steinmann pins on the medial side of the distal femur, along with 2 further Steinmann pins drilled in the anterior-posterior direction (Figures 1 and 3). Via the external fixator, the alignment could be adjusted later by gradually opening or closing the osteotomy. The aim of the osteotomy creation was to adjust the leg axis in 2 different conditions. Neutral alignment (0°) was defined as the mechanical axis of the test rig (from the center of the femoral-sided load cell to the center of the tibial-sided ball-and-socket joint) running through 50% of the distance between the most medial and the most lateral aspects of the knee joint.^10,40^ Varus alignment was defined as the mechanical axis of the test rig crossing the tibial plateau at 25% of the distance between the most medial and the most lateral aspects of the knee joint (Figure 3) corresponding to 5° of varus.^10,40^ The crossing point of the mechanical axis with the tibial plateau was confirmed using the cable method.^6,55^ To adjust for the missing hip and ankle joint, the cable was spun from the center of the femoral fixation to the load cell to the center of the tibial-sided ball-and-socket joint to create a mechanical axis of the test rig. For each specimen, the osteotomy was individually adjusted based on the preexisting mechanical axis. In cases of constitutional valgus, the osteotomy was gradually closed for stepwise neutralization/varisation of the mechanical axis. Conversely, in cases of a preexisting varus deformity, the alignment was adjusted to neutral and the targeted varus of 25% – by opening or closing the osteotomy, depending on the severity of the constitutional varus. After tightening of the external fixation in the specified alignment, any remaining gap of the osteotomy was filled using custom-made 3-dimensional printed wedges to stabilize the osteotomy.

### Sequential Cutting and Reconstruction Protocol

**Figure 4.**
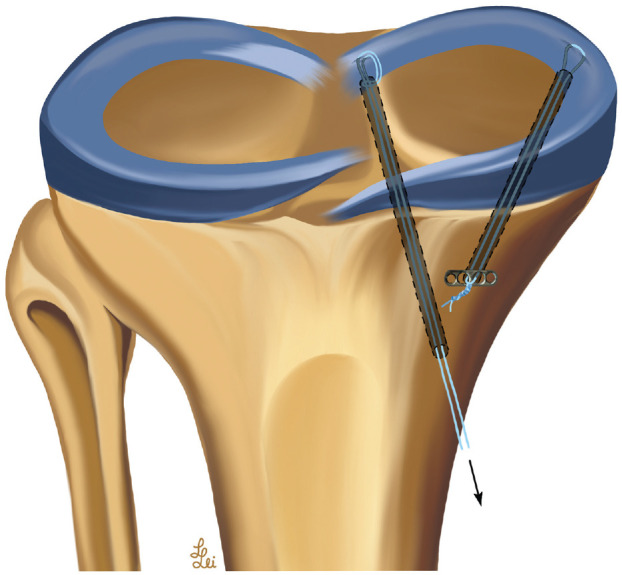
Anatomic medial meniscus root repair with centralization according to Daney et al.^9^ The sutures of the root repair were connected to a custom-made tensioning device to capture the root forces in response to the different testing conditions.

After evaluating the forces on the PMMRR (root repair) in both neutral and varus alignment, the medial meniscus was completely detached from its PA (MTL and MCA) using a scalpel, leaving the joint capsule intact (root repair + PA cut). After repeating the force measurements of the PMMRR, again in neutral and varus alignment, an arthroscopic centralization of the medial meniscus was performed, as previously described (root repair + PA cut + centralization) (Figure 4).^9^ Using a tibial aiming guide (Karl Storz), a 2.0-mm K-wire was placed at the rim of the tibial plateau, at the intersection between the posterior horn and intermediate part of the medial meniscus, and overdrilled using a 4.5-mm cannulated drill bit up to the subchondral bone to avoid damage to the articular cartilage. Care was taken to prevent tunnel collisions between the PMMRR and the centralization tunnel. A horizontal mattress suture with a high-strength polyethylene suture (No. 2 FiberWire) was created using a suture passer (FIRSTPASS MINI). The free ends of the suture were shuttled through the tunnel and knotted over a cortical fixation button (Flipptack; Karl Storz).

### Data Acquisition and Analysis

Data acquisition from the load cell was performed using a custom-made LabVIEW script (LabVIEW 2020). The change of force from the 20-N preload applied to the PMMRR was calculated. Statistical analysis was performed using Prism (Version 9; GraphPad Software). Descriptive data are presented as mean value with standard deviation, and between-group differences are presented as mean difference with 95% confidence interval. The normality of data distribution was verified using histograms and the Shapiro-Wilk test. Mixed linear models with Geisser-Greenhouse correction were used to assess the main effects and interactions of each independent variable (cutting state and flexion angle) for neutral and varus alignment. The dependent variable was change of force at the PMMRR. Pairwise comparisons were used to compare the cutting states at different flexion angles as well as the alignment states in different flexion angles. Post hoc Dunnett correction was performed to account for multiple testing. A *P* value of <.05 was deemed to identify significant differences.

An a priori power analysis was performed using G*Power 2 software.^15^ Based on mean values and standard deviations from a previous study,^37^ it was assumed that a sample size of 8 would allow identification of changes in PMMRR force of 6 N with a SD of 4 N (effect size/Cohen *d* = 1.5), with 95% power, at the significance level of *P* < .05.

## Results

### Influence of Peripheral Attachments and Centralization

**Figure 5.**
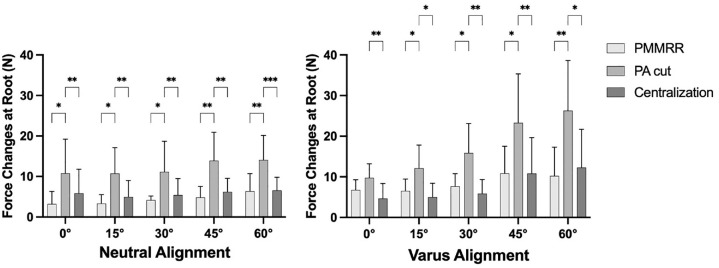
Force changes at a posterior medial meniscus root repair (PMMRR) due to dynamic varus loading, shown as mean value and SD. Centralization, transosseous arthroscopic meniscal centralization; PA cut, cut of peripheral attachments (meniscotibial and meniscofemoral ligament). *P < .05; **P < .01; ***P < .001.

Force changes at PMMRR, due to dynamic varus loading, are shown in Figure 5 for neutral and varus alignment, as well as for each stabilization stage separately.

In neutral alignment, axial compression caused a change of force at the PMMRR from 3.1 ± 3.1 N in 0° flexion to 6.3 ± 4.4 N in 60° flexion (Figure 5, left). After cutting the PA from the medial meniscus (PA cut), the forces increased significantly in all tested flexion angles from 7.0 N (95 % CI 1.0 – 13.0 N; *P* = 0.02) to 9.1 N (95 % CI 4.1 – 14.1 N; *P* = 0.003), in comparison with the intact state (*P* < .05). An arthroscopic centralization (centralization) of the medial meniscus significantly decreased the forces in all flexion angles in comparison with the PA cut state (*P* < .05). No significant differences between the PMMRR and centralization step were found.

In varus alignment, axial compression caused a change of force at the PMMRR from 6.6 ± 2.5 N in 0° flexion to 10.2 ± 7.1 N in 60° flexion (Figure 5, right). In the PA cut, the forces increased significantly, from 5.6 N (95 % CI 0.8 – 10.4 N; *P* = 0.02) in 15° to 16.0 N (95 % CI 5.4 – 26.8 N; *P* = 0.008) in 60° in comparison with the intact state (*P* < .05). Centralization of the medial meniscus significantly decreased the forces in all flexion angles in comparison with the PA cut state (*P* < .05). No significant differences between the PMMRR and centralization step were found.

### Influence of Varus Alignment

**Figure 6.**
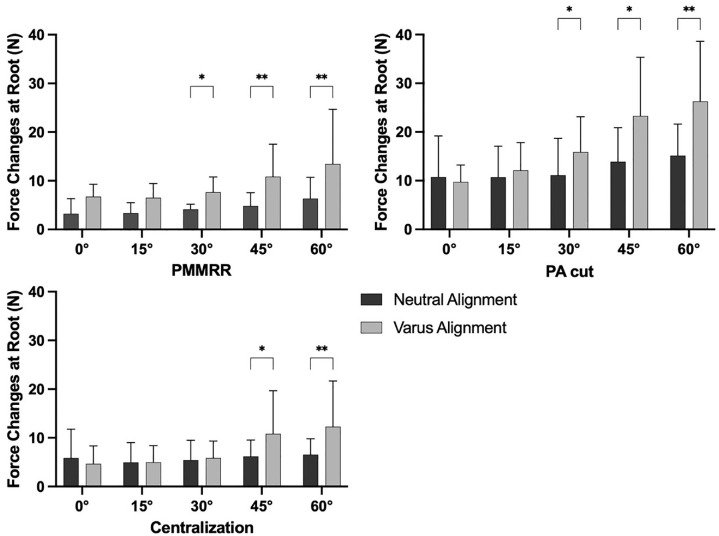
Force changes at a posterior medial meniscus root repair (PMMRR) due to dynamic varus loading in neutral and varus alignment in different cutting states, shown as mean value and SD. Centralization, transosseous arthroscopic meniscal centralization; PA cut, cut of peripheral attachments (meniscotibial and meniscofemoral ligament). *P < .05; **P < .01.

When comparing the alignment states, the varus alignment significantly increased the forces at the PMMRR from 30° (3.5 N; 95% CI, 1.1-5.8; *P* = .01) to 60° (7.1 N; 95% CI, 2.7-11.5; *P* = .007) flexion in comparison with the neutral alignment state (Figure 6). After cutting the PA, the forces at the PMMRR were again significantly higher in varus alignment from 30° (4.8 N; 95% CI, 1.0-8.5; *P* = .02) to 60° (11.1 N; 95% CI, 4.2-18.0; *P* = .006) flexion in comparison with the neutral alignment state. After performing an arthroscopic centralization, a varus alignment caused significantly increased PMMRR forces in 45° (4.7 N; 95% CI, 1.2-8.2; *P* = .01) and 60° (5.8 N; 95% CI, 2.3-9.3; *P* = .002) flexion.

## Discussion

The most important finding of this study was that an insufficiency of the PA of the medial meniscus (MTL and MCA) led to increased forces acting on a PMMRR in both neutral and varus alignment, which was redressable via an arthroscopic meniscal centralization. Furthermore, varus alignment significantly increased the forces on a PMMRR from 30° to 60° knee flexion compared with neutral alignment, which could be a reason for a higher failure rate of medial meniscus root repairs in varus knees.

Root tears of the meniscus are known to lead to an increase in cartilage loading, which subsequently leads to progression of osteoarthritis if untreated.^1,30,36,41,44^ An arthroscopic repair, if possible, is therefore the treatment of choice in patients with a root tear and absence of high-grade osteoarthritis or severe malalignment of the leg.^4,32^ However, high failure rates are described for root repairs of both the medial and lateral menisci. Risk factors for poor postoperative clinical outcome and retear rate include advanced age, high body mass index, and malalignment of the affected leg.^8,20,60^ Furthermore, the concept of meniscal extrusion has recently been recognized as a pathological finding associated with meniscal dysfunction, increased cartilage pressure, and early arthritic changes, as well as an increased failure rate of root repairs and meniscal allograft transplantations.^5,7,8,27,33,35,42^ Additionally, not only root tears but also any type of meniscal tears that cause meniscal hoop dysfunction or meniscal resection could lead to extrusion.^13,18,45^ Recently, however, a clinical study reported on patients with meniscal extrusion in which the meniscal body and roots were found to be intact.^28^ In these patients, signal alterations of the MTL were found, indicating a role in the centralization of the menisci. Furthermore, serial magnetic resonance imaging studies in patients with meniscal extrusion and MTL abnormality have shown subsequent occurrence of PMMR tears.^31^ Based on these findings, it has been postulated that MTL insufficiency is a precursor of PMMR tear because of the increased loads on the roots and that an additional centralization using either transtibial pull-out sutures or knotless suture anchors during arthroscopic root repair restores the load-distributing function of the medial meniscus.^2,9,31^ This theory is supported by the results of the present study, which showed increased forces acting on the PMMRR, after transection of the MTL, in both neutral and valgus alignment, which might lead to pathological loading of the meniscus over time.

The possible influence of the PA on meniscal biomechanics has been previously investigated by several studies, focusing mainly on extrusion as the outcome parameter. In 2 biomechanical studies on human cadaveric knee specimens utilizing a material testing machine, cutting of the MTL led to a significant increase in extrusion of the medial meniscus from 1.5 ± 0.6 mm to 3.4 ± 0.7 mm.^11,46^ Another study, investigating medial meniscal extrusion in human cadaveric knee specimens using a material testing machine, found that transection of the medial meniscus posterior root increased meniscal extrusion and peak contact pressures in the medial compartment.^9^ One biomechanical study, using cadaveric knee specimens in a material testing machine, investigated the influence of the PA on meniscus root forces, measured using a 3-axis sensor.^38^ Cutting the MTL was found to increase forces at the PMMR (no mean differences reported) under compression. A centralization, utilizing a single knotless suture anchor, was not able to restore native forces at the root. Conversely, in the present study, a transosseous centralization was able to restore the root forces back to the PA intact state in both neutral and varus alignment. The differences between the 2 studies might be attributable to differences in the test rig (no dynamic varus possible vs dynamic varus possible) as well as acquisition of root forces (3-axis sensor vs load cell). Finally, in a recent biomechanical study utilizing cadaveric knee specimens, an increased posterior tibial slope as well as an increasing flexion angle of the knee was also found to increase the forces acting on the PMMR, indicating an influence of lower extremity alignment on meniscus root repairs.^37^ Additionally, the present study found that a varus alignment of the knee leads to increased PMMRR forces, irrespective of the cutting state.

This study is of clinical relevance because of its possible implications for the treatment of meniscal pathologies. Based on the findings, an arthroscopic meniscal centralization should be considered in cases of root repair in which the pathology of the PA is evident on magnetic resonance imaging or during arthroscopy. Indeed, the first studies investigating arthroscopic centralization as an adjunct for PMMRRs reported favorable results.^29,39^ Furthermore, realigning femoral or tibial osteotomies can be considered to reduce the load on a PMMRR, as varus malalignment has been shown to result in increased forces on a root repair. In this context, a recent retrospective study reported promising short-term clinical and radiological results after combined valgus-producing high tibial osteotomy and meniscal centralization in varus knees with medial meniscal extrusion.^22^ However, high-quality clinical trials comparing root repair with and without centralization and/or realigning procedures are currently lacking.

Naturally, this study had notable limitations. As with all biomechanical studies, this was a time-zero study that could not reflect the healing effect of an MTL lesion over the course of rehabilitation. Furthermore, cadaveric knee specimens of older age (mean age, 73.0 ± 9.5 years) were used for biomechanical testing, which might not necessarily reflect clinical reality because root repair is typically performed in younger patients. Whether preexisting laxity of the MTL/MCA was present in the tested specimen could not be determined. However, none of the specimens had a degenerative posterior meniscus root tear, making this confounder unlikely, as isolated pathologies of the PA have been described as rare.^28^ The force changes at the PMMRR, as shown in the present study, were comparably small, which might limit the clinical relevance of the findings. However, the loading conditions in the present study were subphysiological (200 N) to allow repeated testing without destruction of the osteotomy or specimen. In previous biomechanical investigations of the medial meniscus roots in a dynamic knee joint simulator simulating physiological muscle loading of the knee joint, the strains on the meniscus tripled after application of muscle forces, with loads up to 140 N.^49-51^ This reinforces that the effects observed in the present study will probably be exaggerated in the physiological environment.

In this study, the medial meniscus was completely detached from its PA, simulating the instability pattern during meniscal allograft transplantation rather than meniscal repair.^14,28,31,35^ Moreover, the present study used a transosseous root repair and centralization. The centralization sutures were tied over a cortical button, which might elongate, possibly influencing the results. Several different techniques exist for arthroscopic centralization of the menisci, ranging from transtibial pull-out sutures^9^ to techniques using from 1 to 3 suture anchors.^2,26,34^ How these different techniques could affect the forces acting on the roots could not be investigated in the present study. Furthermore, the resulting medial meniscal extrusion and changes in knee joint kinematics were not assessed.

Finally, determination of the leg axis was performed by determining the intersection of the mechanical axis of the test rig with the tibial plateau, which might lead to small inaccuracies in comparison with determining joint angles on long-leg radiographs.^5,9^

## Conclusion

An insufficiency of the PAs of the medial meniscus, as well as varus alignment, led to increased forces on a PMMRR. The effect of an insufficiency of the PAs was redressable via arthroscopic meniscal centralization.
